# Phosphorus losses from monitored fields with conservation practices in the Lake Erie Basin, USA

**DOI:** 10.1007/s13280-014-0624-6

**Published:** 2015-02-15

**Authors:** Douglas R. Smith, Wendy Francesconi, Stan J. Livingston, Chi-hua Huang

**Affiliations:** 1 USDA-ARS, Grassland, Soil and Water Research Laboratory, 808 East Blackland Road, Temple, TX 76502-6712 USA; 2 USDA-ARS, National Soil Erosion Research Laboratory, 275 South Russell St., West Lafayette, IN 47906 USA

**Keywords:** Blind inlets, Conservation practices, Crop rotation, Grassed waterway, No-tillage, Water quality

## Abstract

Conservation practices are implemented on farm fields in the USA through Farm Bill programs; however, there is a need for greater verification that these practices provide environmental benefits (e.g., water quality). This study was conducted to assess the impact of Farm Bill eligible conservation practices on soluble P (SP) and total P (TP) losses from four fields that were monitored between 2004 and 2013. No-tillage doubled SP loading compared to rotational tillage (e.g., tilled only before planting corn); however, no-tillage decreased TP loading by 69 % compared to rotational tillage. Similarly, grassed waterways were shown to increase SP loads, but not TP loads. A corn–soybean–wheat–oat rotation reduced SP loads by 85 % and TP loads by 83 % compared to the standard corn–soybean rotation in the region. We can potentially attain TP water quality goals using these Farm Bill practices; however, additional strategies must be employed to meet these goals for SP.

## Introduction

In the United States, agricultural expenditures by the federal government are a part of legislation known as the Farm Bill, which is generally renewed every five years. In the 2008 Farm Bill, $57.7 thousand million was set aside for spending on conservation programs. This is equivalent to $37.38 for every resident of the USA. These expenditures are used by United States Department of Agriculture (USDA) agencies, such as the Natural Resources Conservation Service (NRCS), Farm Service Agency and Forest Service, to pay farmers for financial (i.e., payments made to share the cost of implementing practices) or technical assistance (i.e., payments made to share the cost of designing structural practices).

Lake Erie is the southern most of the five great lakes that form a portion of the border between USA and Canada. Lake Erie is biologically the most active of the great lakes, and as such the charter fishing industry in the state of Ohio, USA has grown to more than $1 thousand million year^−1^. In recent years, harmful and nuisance algal blooms (HNABs) have become more prevalent with the predominant contributing factor being the mass of soluble P loading to the lake during the March to June timeframe (Davis et al. 2009; Bridgeman et al. 2012; Wynne et al. 2013). Two reports have set a 39 % reduction goal for TP loading to Lake Erie compared to the 2007–2012 average (International Joint Commission 2013), and a 37 % decrease in the spring (March through June) loads (Ohio Phosphorus Task Force 2013).

The Maumee River is the largest tributary of the Western Lake Erie Basin, and drains 19 940 km^2^. The St. Joseph River watershed is 2810 km^2^ and represents the headwaters of the Maumee River. Some of the primary Farm Bill conservation practices that have been adopted in the St. Joseph River watershed from 2005 to 2013 are presented in Table 1. The relevant practices to the current study include: conservation crop rotation; residue and tillage management, no-till; grassed waterway; underground outlet; and water and sediment control basin. The conservation crop rotation was the most adopted practice in terms of number of contracts and the area impacted within the watershed. Roughly 5.8 % of the watershed was enrolled in conservation crop rotation and 5.8 % was also enrolled in no-till through Farm Bill programs during this period. There was a total of 101 new contracts for grassed waterways during this period, in which 26 ha of the practice was installed which treated 733 ha. It should be noted that many of these practices existed in the watershed prior to 2005, so many of these practices are more common throughout the St. Joseph River watershed than Table 1 would indicate; however, information in this table represents the period for which electronic records have been kept for adoption of these practices.

**Table 1:** List of some of the conservation practices installed in the St. Joseph River Watershed from 2005 to 2013. This timeframe represents the period for which electronic records were kept and can be queried. The area impacted represents the sum of the field sizes where the practice was applied. The column for installed represents how USDA reports adoption of the practice (note, units for adoption can be area, length or number of practices installed)

Conservation practice	Contracts	Area impacted (ha)	Installed^a^	Units
Conservation crop rotation	1418	16 418	16 418	ha
Residue and tillage management, No-till	1408	16 366	16 366	ha
Nutrient management	959	11 318	11 318	ha
Integrated pest management	712	8503	8453	ha
Conservation cover	541	3315	2976	ha
Filter strip	309	1928	282	ha
Cover crop	232	2888	2720	ha
Grassed waterway	101	733	26	ha
Underground outlet (Blind inlet)	17	361	6095	m
Water and sediment control basin	10	306	25	no

^a^The “installed” column represents the area (or length for underground outlet and number for water and sediment control basin) that was installed in the St. Joseph River watershed. This number may differ slightly than the “area impacted.” For example, there were 101 contracts in the watershed to install a total of 26 ha of grassed waterway. The sum of the drainage area to grassed waterways (i.e., the area impacted) was 733 ha

No-till may be expected to impact P losses, as this practice has been promoted since the 1980s by conservation groups to decrease sediment loss from fields. Similarly, grassed waterways are installed in areas where water can seep from soils and thereby cause ephemeral gully erosion. It is therefore expected that grassed waterways may impact P loading. Conservation crop rotation is currently being promoted by USDA-NRCS as one component of a program to promote soil health. The concept is that introducing more crops into the rotation will improve overall soil quality and the “healthier” soil will decrease sediment, N and P losses from fields. Further, with crops that are harvested in early summer, such as wheat and oats, there is more time to apply fertilizers during a period of low hydrologic activity and thereby lower probability of losing P from applied fertilizers through runoff. Tile risers only offer filtering out large debris, as drainage occurs through holes that are between 1 and 2.5 cm in diameter. The blind inlets may be expected to decrease sediment and nutrient loadings through more tortuous pathways that the ponded runoff water will have to move through in order to be transported out of the field. With the biologic and economic importance of Lake Erie, it is imperative that agriculture in the region utilizes conservation practices to minimize P loading. The objectives of this research were to compare (a) no-tillage, (b) conservation crop rotation, (c) grassed waterways, and (d) blind inlets to the conventional agricultural practices in the St. Joseph River watershed using data from four fields that have been monitored.

## Materials and methods

### Site description

In 2002, the NRCS of USDA was tasked with quantifying the environmental benefits of Farm Bill conservation spending programs. This resulted in collaboration between NRCS and the USDA Agricultural Research Service (ARS) to initiate the Conservation Effects Assessment Project (CEAP), which has a goal of identifying the impacts of conservation programs on water, air and soil quality.

**Fig. 1:**
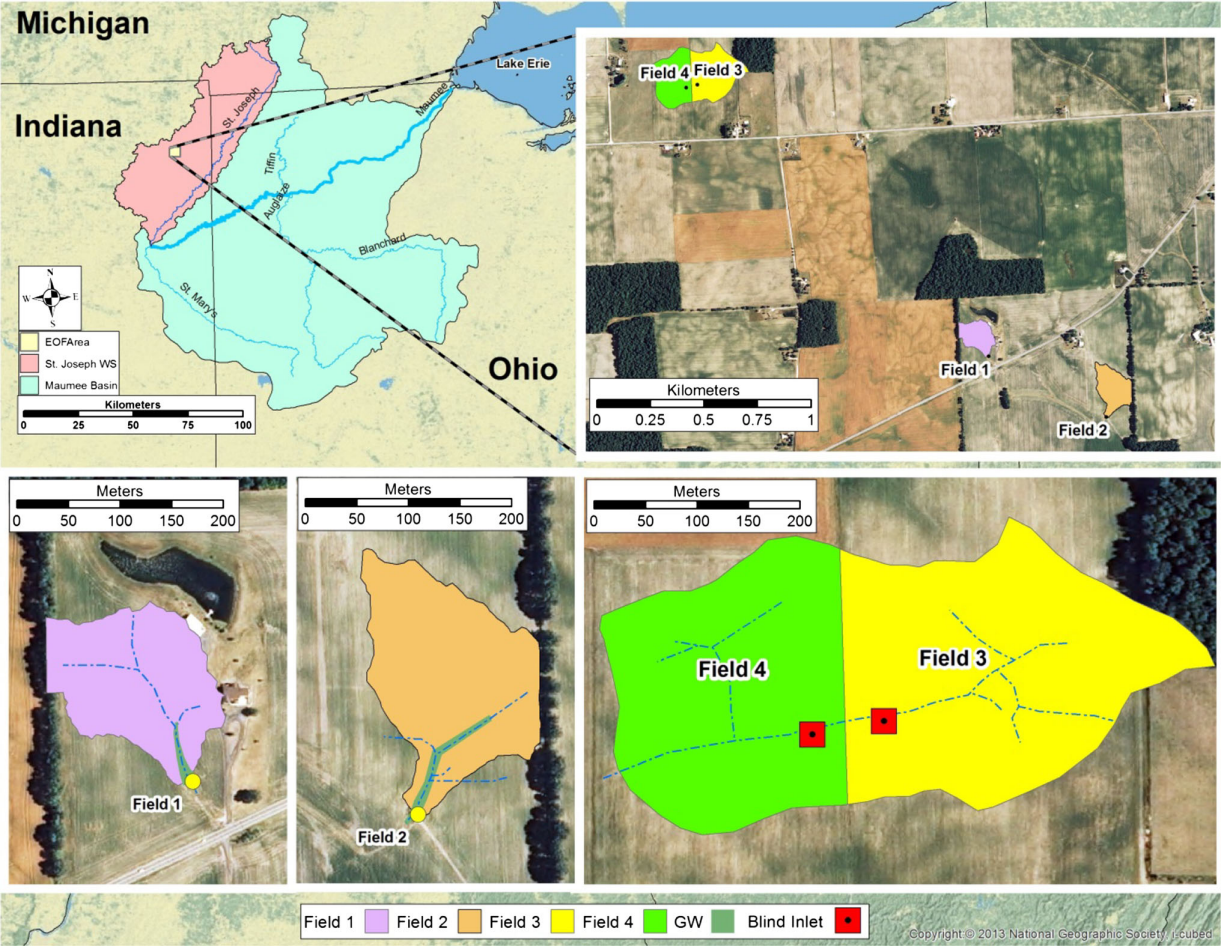
Map showing the location of the four fields in the St. Joseph River watershed and Maumee River Basin, in northeast Indiana, USA, where monitoring was conducted. Note the following abbreviations: edge-of-field (EOF); grassed waterway (GW); and watershed (WS)

In 2002, the St. Joseph River watershed, located in northeast Indiana, USA, was selected for monitoring to determine if implementing voluntary best management practices could result in reduced pesticide loading to drinking water or reduced nutrient loading to the downstream water bodies, including Lake Erie. Four fields in the St. Joseph River watershed have been monitored for surface runoff and tile discharge (Fig. 1; Table 2). These fields were privately owned, so the primary selection criteria were a portion of the area in a single management zone draining surface runoff to a single point, and that the land owner be willing to allow access for long-term monitoring. Fields 1 and 2 are owned by one farmer and managed with a corn/soybean rotation. Surface runoff monitoring in fields 1 and 2 began in 2004, with the installation of drop box weirs at locations that drain 2.2 and 2.7 ha, respectively. Field 2 is 0.6 km southeast of field 1. Both fields had been in no-tillage since approximately 1990. No-tillage was maintained in field 1 throughout the duration of this study. Rotational tillage in field 2 began in 2004, immediately prior to the planting of the corn crop, and continued in corn years until the end of the monitoring period represented in this study. Surface drainage occurs in these fields through dendritic flowpaths that exit the field. Tile discharge monitoring in these fields began in 2008. Discharge measurements were recorded every 10 min and a sample of surface runoff was collected every 30 min when present. Subsurface tile discharge was recorded every 2 min and tile water samples were collected every 90 min. Water samples were refrigerated immediately upon collection, and removed from autosamplers within 48 h of collection.

**Table 2:** Properties of fields in the St. Joseph River Watershed located in northeast Indiana, USA

Property	Field 1	Field 2	Field 3	Field 4
Size (ha)^a^	2.2	2.7	4.0	3.5
Soil series	Glynwood loam Pewamo silty clay Morley silty clay loam	Blount silt loam Glynwood loam	Glynwood loam Pewamo silty clay Wallkill silt loam	Glynwood loam Morley silty clay loam Wallkill silt loam
Soil taxonomy of dominate soil series	Fine, illitic, mesic Aquic Hapludalfs	Fine, illitic, mesic Aeric Epiaqualfs	Fine, illitic, mesic Aquic Hapludalfs	Fine, illitic, mesic Aquic Hapludlalfs
Mehlich 3 P (mg kg^−1^)	28	33	18	22
Depth to tile (m)	0.9	0.9	0.9	0.9
Tile diameter (cm)	10	10	15	10
Flowpath	Dendritic	Dendritic	Closed depression	Closed depression
Tillage	No-tillage	Rotational tillage^b^	Conventional tillage	Conventional tillage
Crop rotation	Corn/soybean	Corn/Soybean	Corn/soybean/oat/wheat	Corn/soybean/oat/wheat
Grassed waterway	Installed in 2006	Installed in 2010	–	–
Surface water drainage	–	–	Tile riser/blind inlet	Tile riser/blind inlet

^a^Field size in this context refers to the portion of the field that drains to the monitoring point. Site selection required a portion of a single management unit to drain to a single point for monitoring. The fields as managed by the farmers were larger than the drainage area

^b^Rotational tillage in field 2 occurred using a chisel/disk in one pass within 3 days prior to planting. Depth of tillage was approximately 10 cm

Fields 3 and 4 are a pair of closed depressions, locally known as potholes, located 1.88 km northwest of field 1 and are owned by one farmer. Traditionally, ponded runoff water in fields like these is drained with tile risers. We have developed a practice, called blind inlets, to drain the ponded surface water and provide greater treatment than tile risers, (for more details, see Smith and Livingston 2013). Briefly, tile risers that were installed in each closed depression were plastic 15-cm-diameter pipe that extended approximately 1 m above the soil surface, and had approximately 2 cm diameter holes to drain water while retaining large debris. The blind inlets were constructed by removing 4.25 × 4.25 × 1.0 m of soil. Rigid plastic 10-cm-diameter septic tile line was placed on 10 cm of limestone gravel (approximately 3–5 cm diameter) bed in the hole, followed by placing another 60 cm of limestone gravel. A geotextile fabric (Typar 3301; Fiberweb, Old Hickory, TN, USA) was used to blanket the gravel layer and a 30-cm course soil material was used to bring the excavated area to the same elevation as the adjacent soil. Gate valves were used to allow drainage of ponded water with either the blind inlet or the tile riser in both fields. Blind inlets are novel, because they allow farmers to perform field operations over the drainable area, unlike tile riser. Both field 3 and 4 were instrumented with a tile riser and a blind inlet, so that both practices could be tested in either field. Generally, the tile riser drained one field while the blind inlet drained the other; however, periodically the drainage treatment within each field was switched. Surface runoff and tile discharge were monitored and samples collected similar to the tile discharge for fields 1 and 2.

### Treatments and data

The treatments that have been applied to these fields are presented in Table 3. In short, field 1 was cropped using continuous no-tillage (planting directly into crop residues from previous crop), field 2 was converted from continuous no-tillage to rotational tillage (tilled prior to planting corn), and field 3 and 4 were tilled between each crop. Fields 1 and 2 were cropped using a corn/soybean rotation with corn planted in even numbered years, while fields 3 and 4 were cropped using a corn/soybean/wheat/oat rotation (here referred to as conservation crop rotation). Ephemeral gulleys developed in field 1 during the winter of 2005/2006 and in field 2 during the winter of 2009/2010, thus grassed waterways were established in these fields in 2006 and 2010, respectively, to address this resource concern. As mentioned above, fields 3 and 4 were equipped with both a tile riser and a blind inlet, and when one field was tested with the blind inlet the other field was tested with the tile riser as a control.

**Table 3:** Crops, conservation practices in each field and the number of surface runoff events from the four fields monitored in the St. Joseph River watershed

	Field 1			Field 2			Field 3			Field 4		
	Crop	Practices^a^	Events	Crop	Practices	Events	Crop	Practices	Events	Crop	Practices	Events
2004	Corn	N	12	Corn		14						
2005	Soybean	N	1	Soybean		4	Alfalfa	N, C	1	Corn	C	6
2006	Corn	N, G	6	Corn		18	Alfalfa	N, C	7	Soybean	C	8
2007	Soybean	N, G	6	Soybean		8	Wheat	C, B	3	Wheat	C, B	4
2008	Corn	N, G	2	Corn		0	Corn	C	3	Silage	C, B	3
2009	Soybean	N, G	9	Soybean		16	Soybean	C, B^b^	4	Soybean	C, B^b^	4
2010	Corn	N, G	10	Corn	G	12	Oats	C	7	Oats	C, B	9
2011	Soybean	N, G	11	Soybean	G	13	Wheat	C, B	10	Wheat	C	10
2012	Corn	N, G	0	Corn	G	1	Corn	C, B	0	Corn	C	0
2013	Soybean	N, G	7	Soybean	G	4	Soybean	C	8	Soybean	C	8

^a^Abbreviations for Conservation Practices: *N* no-till, *G* grassed waterway, *C* conservation crop rotation, *B* blind inlet

^b^In 2009, there were events in fields 3 and 4 on 3 April, 5 April, 23 October, and 30 October. The blind inlet was used in field 4 for the April events and in field 3 for the October Events

Monitoring data represents the “growing season” which extends from April 1 to November 15 of each year. In years with good weather, April 15 is the target date to plant corn and soybean, while soybean harvest typically begins in the latter half of September and corn harvest in October. This project was initiated as a pesticide project, and as such the sampling protocols were developed with pesticides (and periods when pesticides might be expected to be applied to the landscape) as the primary constituent.

### Sample analysis

All nutrient analyses were conducted colorimetrically with a Konelab Aqua 20 (EST Analytical, Medina, OH). Soluble P (SP) was analyzed on the vacuum filtered (0.45 µm; Fisher Scientific) acidified samples using EPA method 365.2 (U.S. EPA 1983). Total P was analyzed using EPA method 365.4 (U.S. EPA 1983) after mercuric sulfate digestion of the unfiltered samples.

### Statistics

The statistical design used for this experiment was the before-after/control impacted. Flow-weighted mean (FWM) concentrations for surface runoff were calculated for each runoff event based on the total mass of contaminant lost and the total volume of water discharged. Comparison of medians was made using JMP v 10.0.0 (SAS Institute Inc.) with 0.10 set as the level to establish significant differences. Conservation practices present in each field and the number of surface runoff events from each field are presented in Table 3. Tillage comparisons were made using field 1 as the no-tillage treatment, field 2 as the rotational tillage treatment and fields 3 and 4 as the tilled treatment. Conservation crop rotation was tested by comparing fields 3 and 4 to fields 1 and 2 which were farmed with the conventional corn–soybean rotation. For comparison of grassed waterways, data from fields 1 and 2 were used, with 2004–2005 used as the control period for field 1 and 2004–2009 as the control period for field 2. Fields 3 and 4 were used to compare the impact of the blind inlet to the tile riser on P loading. The loads given represent the growing season, as the April 1 to November 15 period of each year is when water quality samples were collected.

## Results

### Hydrology

**Fig. 2:**
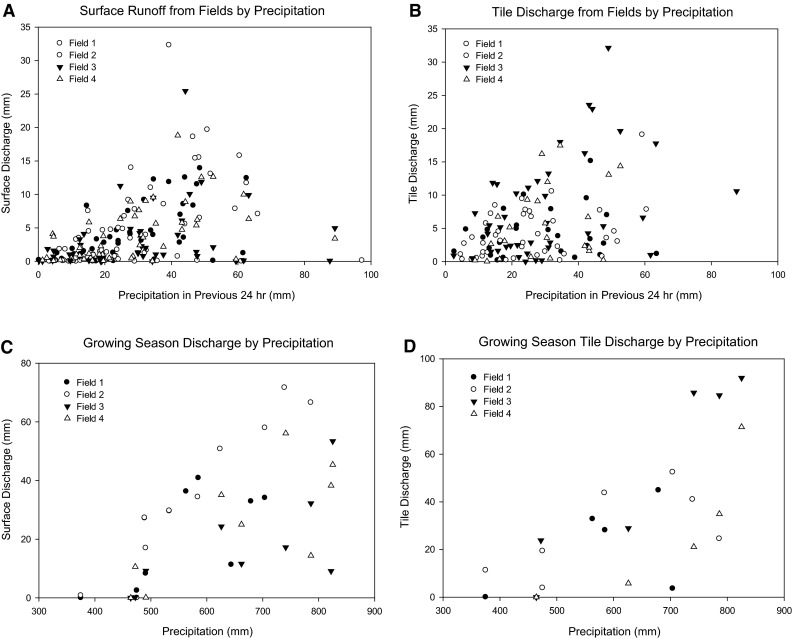
Surface and tile discharge graphed against precipitation. **a** surface runoff from fields for individual storm events, **b** tile discharge from fields for individual storm events, **c** surface runoff from fields for the entire growing season (April 1–November 15 each year), and **d** tile discharge from fields for the growing season (April 1–November 15 each year)

Growing season precipitation ranged from 375 mm to 825 mm (Fig. 2). Median precipitation was 548 mm for field 1, 559 mm for field 2 and 662 mm for fields 3 and 4, which shared a rain gauge. There were 64, 89, 45, and 54 surface runoff events for fields 1, 2, 3, and 4, respectively (2004–2013), and 27, 43, 41, and 23 tile flow events (2008–2013), respectively, from these fields. There were no significant differences in surface runoff or tile discharge from fields resulting from any of the conservation practices implemented in this study.

### Conservation tillage

**Fig. 3:**
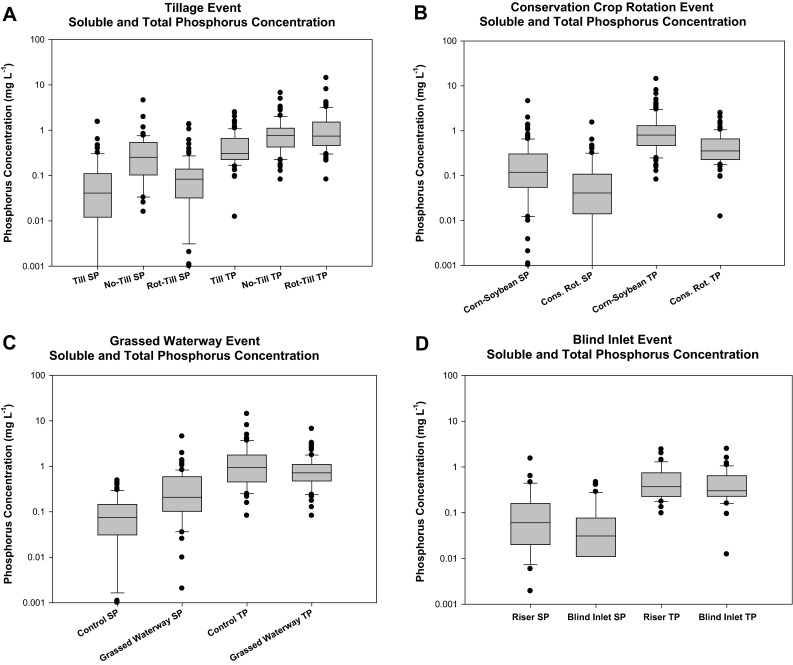
Median soluble P (SP) and total P (TP) FWM concentrations in surface runoff during individual storms by **a** tillage practice; **b** crop rotation; **c** grassed waterway; and **d** blind inlet. Note the following abbreviations: tillage (Till); rotational tillage (Rot–Till); corn–soybean rotation (Corn–Soybean); conservation rotation (Cons. Rot.); and tile riser (Riser)

Median FWM concentrations of SP during individual surface runoff events were significantly greater (*p* < 0.001) for no-tillage (0.25 mg L^−1^) compared to either rotational tillage (0.08 mg L^−1^) or the tilled system (0.04 mg L^−1^; Fig. 3a). No-tillage and rotational tillage had greater (*p* < 0.001) median FWM TP concentrations than the tilled fields.

**Fig. 4:**
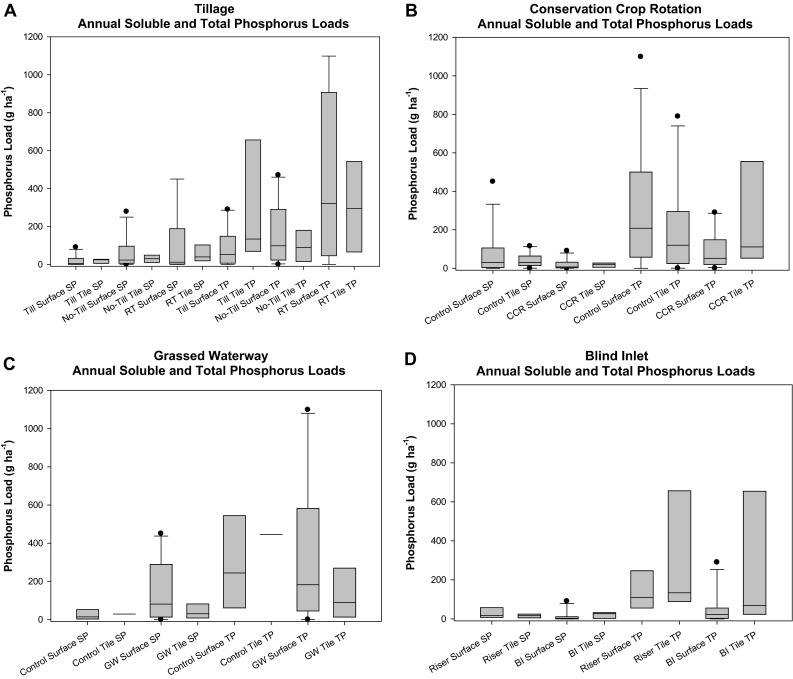
Growing season (April 1–November 15) soluble P (SP) and total P (TP) loads by **a** tillage practice; **b** crop rotation; **c** grassed waterway; and **d** blind inlet. Note the following abbreviations: tillage (Till); rotational tillage (RT); corn–soybean rotation (Control); conservation rotation (CCR); grassed waterway (GW); tile riser (Riser); and blind inlet (BI)

A growing season surface runoff SP load of 22.9 g ha^−1^ from the no-tillage field was greater (*p* < 0.10) than for the rotational tillage (11.6 g ha^−1^) or the tilled (5.1 g ha^−1^) treatments (Fig. 4a). However, the rotational tillage treatment (321 g ha^−1^) resulted in significantly greater (*p* < 0.001) growing season surface runoff TP loads than either no-tillage (98 g ha^−1^) or tillage (51.7 g ha^−1^).

There was no significant difference between the growing season tile SP load from tilled, no-tillage or rotational tillage treatments (24.5, 30.2, and 39.8 g ha^−1^, respectively; Fig. 4a). Tile TP growing season loads were also not significantly different between tillage treatments.

### Conservation crop rotation

Conservation crop rotation (0.04 mg L^−1^) had lower (*p* < 0.001) median FWM SP concentrations in surface runoff than the corn/soybean rotation (0.12 mg L^−1^; Fig. 3b). Further, the conservation crop rotation (0.36 mg L^−1^) had significantly lower (*p* < 0.001) median FWM TP concentration than the 0.80 mg L^−1^ observed from the corn–soybean rotation.

Soluble P growing season loads of 31 g ha^−1^ from the corn/soybean rotation were greater (*p* < 0.05) than the 4.6 g ha^−1^ observed from the conservation crop rotation (Fig. 4b). Total P growing season loads were also greater (*p* < 0.01) from the corn/soybean rotation (208 g ha^−1^) compared to the conservation crop rotation (41.3 g ha^−1^).

Tile discharge SP growing season loads (Fig. 4b) from the conventional rotation (31.7 g ha^−1^) were slightly greater (p < 0.15) than the conservation crop rotation (24.5 g ha^−1^). There was no significant difference for growing season tile TP loads from the conventional treatment (139 g ha^−1^) and the conservation crop rotation (134 g ha^−1^).

### Grassed waterway

A significant increase (*p* < 0.001) was observed for median FWM SP surface runoff concentrations following the installation of grassed waterways (0.21 mg L^−1^) compared to prior to installation (0.08 mg L^−1^; Fig. 3c). There was a slight decrease (*p* = 0.12) in FWM TP concentrations following the installation of grassed waterways in fields 1 and 2.

Grassed waterway installation significantly increased (*p* < 0.05) growing season surface runoff SP loads from fields 1 and 2 from 14 to 81 g ha^−1^ (Fig. 4c). No significant change (*p* = 0.40) in growing season TP loads were observed when comparing periods prior to grassed waterway installation (224 g ha^−1^) to those after installation (182 g ha^−1^).

Growing season tile discharge SP loads were not significantly impacted by grassed waterway installation (Fig. 4c). Prior to incorporating the grassed waterways, the growing season SP load was 29.1 g ha^−1^ and was 31.7 g ha^−1^ after installation. Similarly growing season tile TP loads were not significantly different between the two treatments. Unfortunately, there were only two datapoints for annual tile discharge without grassed waterways, so we were unable to detect differences for this flow path.

### Blind inlet

When fields 3 or 4 were drained with blind inlets (0.03 mg L^−1^) there was a significant decrease (*p* < 0.05) in median FWM SP surface runoff concentration compared to when these fields were drained using the tile risers (0.06 mg L^−1^; Fig. 3d). There was no significant difference (*p* = 0.59) in median FWM TP concentrations between the tile riser (0.37 mg L^−1^) and blind inlet (0.30 mg L^−1^) surface runoff drainage practices.

The blind inlet decreased (*p* < 0.10) growing season surface SP loads from 17 g ha^−1^ for the tile riser to 2.8 g ha^−1^ (Fig. 4d). Similarly, growing season TP loads were decreased (*p* < 0.10) from 110 g ha^−1^ when fields 3 or 4 were drained with the blind inlet to 23 g ha^−1^ when drained by the blind inlet.

Tile SP growing season loads of 27 g ha^−1^ from blind inlets were not significantly different from median tile SP loads of 16 g ha^−1^ from fields drained with tile risers (Fig. 4d). Tile TP growing season loads from the tile riser drained fields was 134 g ha^−1^, which was not significantly different from the 160 g ha^−1^ growing season TP loads observed for the blind inlet drained fields.

## Discussion

The range of median FWM SP concentrations in surface runoff, from 0.03 to 0.25 mg L^−1^, for the conservation practices monitored in this study was at the lower end of the range reported in literature. In a continuous corn rotation in Ontario, Canada, 0.1 mg L^−1^ SP concentrations were reported for surface runoff (Culley et al. 1983). Algoazany et al. (2007) found surface runoff SP concentrations between 0.25 and 0.57 mg L^−1^ from fields in Illinois, USA. Monitoring of fields in Wisconsin, USA, a range in SP concentrations in surface runoff of 0.67–4.43 mg L^−1^ was reported (Ruark et al. 2012); however, the fields with higher SP concentrations were the result of being heavily manured.

Surface runoff total P concentrations in the literature range from 0.19 to 6.3 mg L^−1^ (Culley et al. 1983; Ruark et al. 2012). Monitoring of conservation practices in the present study found a range of 0.31–0.94 mg L^−1^ in surface runoff.

### Conservation tillage

Gaynor and Findlay (1995) reported greater SP losses from no-tillage (1.02 mg L^−1^) than from tilled fields (0.29–0.55 mg L^−1^). While the values reported for SP loads by tillage in this study were much lower, their data set supports the conclusion that no-tillage may indeed result in greater SP concentrations in runoff than from tilled systems. While they did not report TP losses, they did report sediment concentrations to be reduced by as much as 59 % for no-tillage. In the present study, no-tillage reduced sediment concentrations by as much as 82 % (data not shown). In a study of moldboard plowed and ridge tilled systems, it was noted that SP losses were lower following moldboard plowing, but total P losses were greater from this system (Zhao et al. 2001).

Phosphorus stratification in no-till soils has long been recognized, and one method that has been understood to decrease the resulting P losses has been tillage (Sharpley 2003). In Scandinavia, decreased TP losses with greater SP losses resulting from no-tillage has been shown (Ulen et al. 2010). Tillage has been shown to decreasing the surface enrichment of P in surface soils, and thereby the reducing interactions of surface runoff water with P at the surface and decreasing P runoff (Scharer et al. 2007). Ekholm et al. (1999) also showed decreased SP losses resulting from tillage, while Butler and Haygarth (2007) found lower SP losses in soil boxes that were tilled.

Divergent SP and TP losses have also been shown to occur in the Maumee River (Joosse and Baker 2011). While TP loads are decreasing, some conservationists have blamed the increasing SP loads on the adoption of conservation tillage. However, there is currently insufficient evidence of a causal relationship between adoption of conservation tillage and increasing SP concentrations in the Maumee River.

### Conservation crop rotation

Some studies have identified cropping systems as being more important for P loss than year (Stenberg et al. 2012). The conservation crop rotation had the greatest impact on SP and TP in surface runoff and tile discharge. Including winter wheat in the rotation provides benefits of protecting the soil during winter, compared to the fallow soil during winter for the conventional rotation. Further, crops such as wheat or oat, that are harvested during the summer allow for fertilizer applications to be made during the summer, when the risk of runoff P losses are typically low. The farmer that used the conservation crop rotation also fertilized with small amounts of fertilizer prior to each crop, whereas the corn/soybean farmer applied higher rates of P prior to planting corn, but no P prior to planting soybean. Based on plot scale analysis, the latter fertilization strategy leads to greater P losses than lower rates applied annual when averaged over the rotation (D.R. Smith, unpublished data).

Monoculture cropping systems, such as continuous wheat, have been shown to have greater soluble and total P losses than perennial cropping systems (Smith et al. 1991). Alterations in cropping system management to include ground cover from a cash crop during periods when precipitation and runoff are expected, is an important recommendation for decreasing phosphorus losses from agriculture (Withers and Jarvis 1998). In a comparison of a conventional and an organic crop rotation, no differences in P losses were observed (Torstensson et al. 2006). In a similar study, the conventional rotation was found to have lower TP losses than the organic rotation; however, there were no differences in SP loss (Stenberg et al. 2012). Neumann et al. (2011) observed greater leaching of P in rotations with broad bean, potentially due to greater flow through macropores that formed as a result of the taproot. It is important to note that in the rotations of the current study, a taproot structure (i.e., soybean) was reduced from every other year in the conventional rotation to once every four years in the conservation crop rotation.

### Grassed waterway

The importance of placing grassed waterways in surface runoff pathways within fields is well recognized, as evidenced by their inclusion in Nordic regulatory measures for erosion control (Heckrath et al. 2008), and by their inclusion as a cost-share practice in the US Farm Bill. While these in-field buffers are recognized to reduce sediment and thus P losses, release of SP from sediment deposits or from vegetation is also recognized (Withers and Jarvis 1998). In a grassed waterway receiving runoff from a no-till field in Ohio, USA, there was no decrease in SP observed (Shipitalo et al. 2010). Thus, the increase in SP loads in surface runoff from grassed waterways indicated in this study concurs with the literature.

When shallow flow dominates, buffer strips have been shown to be as much as 61 % effective in reducing P losses (Noij et al. 2013). However, given the design of grassed waterways, concentrated flows occur near the center of the practice, which may account for the lower P trapping efficiency in this study. Since the grassed waterways are, by design, grassed, it is possible that stratification of P near the surface results in greater P losses (Scharer et al. 2007). In a study of buffer systems, Owens et al. (2007) observed that buffers tend to trap the larger soil particles, which can result in enrichment of runoff water with respect to P.

### Blind inlet

Total P loads from the current study were similar to those (ca. 0.1 kg ha^−1^) observed from monitoring of closed depressions in Minnesota, USA (Ginting et al. 2000), however that study reported slightly greater SP loads of roughly 0.05 kg ha^−1^. Using a slightly modified blind inlet design in Minnesota, USA, Feyereisen et al. (2014) also observed decreases in SP with blind inlets compared to tile risers. They did not measure TP; however, median sediment concentrations were reduced by an order of magnitude, which would decrease TP losses.

### Subsurface tile

The lack of differences due to tillage in SP or TP loading via subsurface tile is consistent with other studies (Djodjic et al. 2002; Algoazany et al. 2007). Cropping system alteration has been shown to have minimal effect on subsurface tile losses of P (van Es et al. 2004; Kinley et al. 2007). No studies have identified an impact of grassed waterways on subsurface P transport. This is the first study to report on the impact, or lack thereof, of blind inlets on tile drainage P losses; however, since this is a practice designed to treat the surface water entering discrete surface connections, this result is not surprising.

Early work with tile suggested that little P was transported via this pathway (Kladivko et al. 1991; Brady and Weil 1999). However, more recent work indicates that significant amounts of P (40–50 %) can be transported through subsurface tile (Schoumans and Breeuwsma 1997; King et al. 2014). Recent work in Belgium has shown that P leaching in watersheds occurs quicker than previously recognized (de Bolle et al. 2013). In a study of transport pathway from the fields used in the current study 20–80 % of the P lost was via the tile network (Smith et al. 2014). Hodgkinson and Withers (2007) found that between 31 and 55 % of P loss in three English headwater catchments occurred via tile drainage. None of the conservation practices tested made an impact on concentrations and loads of SP or TP through subsurface tile discharge. Many conservation practices, including no-tillage, grassed waterway, and blind inlets, were primarily designed to minimize erosion from agricultural fields.

### Potential impact of Farm Bill

The potential impact of implementing Farm Bill conservation practices between 2005 and 2013 are presented in Table 4. For this assessment, filter strip was estimated to perform similar to a grassed waterway, as the practice standard for these two are similar. Water and sediment control basins were also included in this analysis, as an underground outlet is part of the installation of this practice and blind inlets are included in the underground outlet practice standard.

**Table 4:** Estimated impact of Farm Bill conservation practices placed in the St. Joseph River watershed based on extrapolated results from monitored fields used in this study. Values (kg) presented here are not absolute values, but are provided to estimate the potential impact on water quality in this watershed from the practices that have actually been placed in the watershed

	Estimated untreated				Estimated with conservation			
	Surface		Tile		Surface		Tile	
	SP	TP	SP	TP	SP	TP	SP	TP
No-till	190	5250	651	4830	375	1600	494	1460
Conservation crop rotation	509	936	520	2280	76	159	402	2200
Grassed waterway	10	164	21	326	59	133	23	102
Filter strip^a^	27	432	56	858	156	351	61	268
Blind inlet	6	40	6	48	1	8	10	61
Water and sediment control basin^b^	5	34	5	41	1	7	8	51

^a^Values for filter strip are based on data from grassed waterway. The filter strip practice standard is similar to the practice standard for the grassed waterway

^b^An underground outlet (practice standard that blind inlet is a part of) is used when a water and sediment control basin is installed. The estimates for this practice are based on results from the blind inlet practice since they are similar

The overall impact of these conservation practices for the growing season is a decrease of roughly 80 kg SP and 4600 kg of TP from surface runoff. If tile discharge is included in the analysis, approximately 340 kg SP and 8800 kg TP per growing season were kept out of the surface water and potentially in the soil profile for future use by crops. From 2008 to 2011, between 45 and 87 % of the TP lost from the Maumee River basin to Lake Erie occurred during the non-growing season (November 16–March 31; data not shown). If this observation holds true at the field scale, and the 8800 kg TP benefit from the Farm Bill conservation programs during the growing season could potentially be 16 000 kg TP year^−1^ or more retained in the field. Study of these practices in this watershed during the non-growing season is needed to confirm this estimate.

Often, a small amount of a watershed provides a disproportionate amount of the water quality impairment. One modeling study found that treating 4–12 % of a watershed could vastly improve the water quality (Kovacs et al. 2012). However, the impact of tile drainage in the WLEB suggests a much greater extent of the watershed is a likely contributor to the P losses. Haygarth et al. (2009) indicated that targeting of conservation practices needed to be based on local site conditions. Further, modeling of conservation practices in New Zealand found that a greater extent of existing technologies needed to be adopted or that additional strategies needed to be developed to attain water quality goals (McDowell et al. 2011). Applying results from this study to the St. Joseph River watershed suggests similar implications, a much greater extent of conservation needs to be adopted or additional strategies need to be developed to attain our water quality goals. De Bolle et al. (2013) suggested greater focus on the specific role P fertilizer plays on water quality impairment should be studied. We agree that this should be a future focus of study.

## Conclusions

Most of the conservation practices applied to fields were developed to decrease sediment loss from fields. While sediment losses were not explored in this paper, when these practices were developed, the common knowledge was that if you stop the sediment you will stop the P. This mindset has been disproven. No-tillage decreased surface runoff TP loads by 223 g ha^−1^ compared to rotational tillage, but SP was nearly double from no-tillage. Soluble P and TP were 34 and 52 g ha^−1^ less in surface runoff and tile discharge from the conservation crop rotation than the corn–soybean rotation. Grassed waterways decreased SP by 67 g ha^−1^ and TP by 42 g ha^−1^ in surface runoff. Blind inlets decreased SP and TP loads in surface runoff by 14 and 87 mg L^−1^, respectively, compared to the tile risers.

Between 2005 and 2013, there were 36 112 ha of conservation practices applied within the 281 232 ha St. Joseph River watershed. On the land base of applied conservation practices, we estimate that SP was decreased from 2010 to 1670 kg P per growing season and TP was decreased from 15 200 to 6400 kg per growing season. This represents a decrease of 17 and 58 % in SP and TP loads, respectively, for the treated acres. Adoption of these practices on many fields predates the 2005–2013 period when we were able to collect these records, so it is difficult to discern how many more acres would need adoption of these practices to achieve the goal of a 39 % decrease in total P loading; however, it does appear that this level could be achieved through adoption of these practices. However, based on the relatively low impact on SP, it does not appear adoption of these practices will achieve the target of a 41 % decrease in SP loading to Lake Erie. Thus, our results concur with other reports in that greater adoption of these practices in addition to new strategies will need to be adopted in order to achieve water quality goals.
